# Supplementary material for: The Rochester Modified Magee Algorithm (RoMMa): An Outcomes Based Strategy for Clinical Risk-Assessment and Risk-Stratification in ER Positive, HER2 Negative Breast Cancer Patients Being Considered for Oncotype DX® Testing
Source: Cancers (Basel). 2023 Jan 31;15(3):903. doi: 10.3390/cancers15030903 (PMC9913115; doi:10.3390/cancers15030903)
Supplement: Supplementary file 1 [file cancers-15-00903-s001.zip › cancers-2108399-supplementary.pdf]

Supplemental table S1: DISCORDANT VERY LOW RISK/HIGH RISK CASES

	PATHOLOGIC CHARACTERISTICS										
**RISK CATEGORY**	**aMMs**	**ODX**	**AGE (years)**	**NS**	**ER***	**PR***	**KI-67**	**SIZE (cm)**	**LVI**	**NODE STAGE**	**FOLLOW-UP (MONTHS)#**
**VERY LOW aMMs/HIGH ODX**											
Case 1	12.5	28	59	6	285	270	5	1.5	N	0(sn)	93
Case 2	10.9	18	47	6	300	300	1	2.0	N	0	66
Case 3	11.3	16	49	5	270	270	3	1.0	Y	1a	71
Case 4	12.4	16	47	4	225	270	10	2.5	N	0	98
Case 5	11.6	18	47	5	270	270	5	1.5	N	0	123
Case 6	11.6	17	48	5	285	285	8	3.3	N	0	137
AVERAGE	11.7	18.8	49.5	5.2	272.5	277.5	5.3	2.0			98.0
**VERY LOW ODX/HIGH aMMs**											
Case 1	18.7	10	73	8	250.5	250.5	25	2	Y	0	63
Case 2	21.7	10	51	7	297	45	20	1.8	N	0	65
Case 3	22.5	9	64	5	270	15	10	11.6	N	1mi	76
Case 4	21.9	4	65	7	285	10	10	1.2	N	0	76
Case 5	19.8	7	47	5	170	80	5	1.4	N	0	78
Case 6	19.2	9	66	6	120	210	7.5	2.9	N	0	79
Case 7	19.0	10	62	5	270	40	7.5	2.5	N	0	81
Case 8	20.8	10	75	6	285	30	10	4	N	1a	84
Case 9	18.7	10	46	3	180	20	5	0.8	N	0	86
Case 10	21.1	9	53	6	240	40	10	2	N	0	87
Case 11	23.6	9	41	6	285	1	15	9	Y	1a	90
Case 12	21.9	2	45	6	140	100	7.5	2.8	N	0	91
Case 13	19.2	6	69	9	270	270	30	1.3	N	0	92
Case 14	22.6	8	68	6	90	90	2	1.5	N	NA	92
Case 15	24.4	5	58	6	180	60	NA**	2.2	N	0(sn)	157
AVERAGE	21.0	7.9	58.9	6.1	222.2	84.1	11.8	3.1			86.5

** Modified H-score[64]

** Not available

Includes total months of follow-up including recurrence

Supplemental table S2: ALL DISCORDANT CASES WITH RECURENCE

	PATHOLOGIC CHARACTERISTICS										
**RISK CATEGORY**	**aMMs**	**ODX**	**AGE (years)**	**NS**	**ER***	**PR***	**KI-67**	**SIZE (cm)**	**LVI**	**NODE STAGE**	**FOLLOW-UP (MONTHS)#**
**LOW aMMs/HIGH ODX**											
Case 1	16.3	19	43	4	285	45	NA**	4	N	0	74
Case 2	16.5	17	50	6	240	240	NA	1.1	N	0	144
AVERAGE	16.4	18.0	46.5	5.0	262.5	142.5	NA	2.6			109
**VERY LOW ODX/HIGH aMMs**											
Case 1	19.2	6	69	9	270	270	30	1.3	N	0	62
Case 2	22.6	8	68	6	90	90	2	1.5	N	NA	18
AVERAGE	20.9	7.0	68.5	7.5	180.0	180.0	16	1.4			40
**LOW ODX/HIGH aMMs**											
Case 1	21.4	14	61	9	285	285	55	2.7	Y	0(sn)	24
Case 2	21.6	15	59	5	120	90	5	4	N	1a	51
Case 3	19.7	13	54	5	300	15	20	0.7	N	0(sn)	126
Case 4	24.6	23	69	7	285	0	NA	2.7	N	0	50
Case 5	21.1	23	67	7	270	70	15	1.5	N	0	69
Case 6	20.9	16	64	9	250.5	250.5	30	3.5	Y	0	82
Case 7	27.2	17	72	6	270	1	60	3.5	Y	1(mi)	96
Case 8	21.1	22	67	5	270	0	10	5.1	N	0	100
Case 9	24.8	18	76	8	300	22.5	30	2.4	N	0	154
AVERAGE	22.5	17.9	65.4	6.8	261.2	81.6	28.1	2.9			83.6

** Modified H-score[64]

** Not available

Includes total months of follow-up including recurrence
